# G6PD Deficiency Is Crucial for Insulin Signaling Activation in Skeletal Muscle

**DOI:** 10.3390/ijms23137425

**Published:** 2022-07-04

**Authors:** Aiwen Jiang, Hongyun Guo, Xiaoyu Jiang, Jingli Tao, Wangjun Wu, Honglin Liu

**Affiliations:** Department of Animal Genetics, Breeding and Reproduction, College of Animal Science and Technology, Nanjing Agricultural University, Nanjing 210095, China; 2019205006@njau.edu.cn (A.J.); guohongyun2022@163.com (H.G.); j0824xy@163.com (X.J.); taojingli@njau.edu.cn (J.T.)

**Keywords:** G6PD, insulin resistance, skeletal muscle

## Abstract

Glucose 6-P dehydrogenase (G6PD) is the first rate-limiting enzyme in pentose phosphate pathway (PPP), and it is proverbial that G6PD is absent in skeletal muscle. However, how and why G6PD is down-regulated during skeletal muscle development is unclear. In this study, we confirmed the expression of G6PD was down-regulated during myogenesis in vitro and in vivo. G6PD was absolutely silent in adult skeletal muscle. Histone H3 acetylation and DNA methylation act together on the expression of G6PD. Neither knock-down of G6PD nor over-expression of G6PD affects myogenic differentiation. Knock-down of G6PD significantly promotes the sensitivity and response of skeletal muscle cells to insulin; over-expression of G6PD significantly injures the sensitivity and response of skeletal muscle cells to insulin. High-fat diet treatment impairs insulin signaling by up-regulating G6PD, and knock-down of G6PD rescues the impaired insulin signaling and glucose uptake caused by high-fat diet treatment. Taken together, this study explored the importance of G6PD deficiency during myogenic differentiation, which provides new sight to treat insulin resistance and type-2 diabetes.

## 1. Introduction

Skeletal muscle, which makes up over 40% of body weight, is the largest organ in the body [1]. In livestock, the development of skeletal muscle largely determines the body growth and meat production [2,3]. As the protein reservoir of mammals, skeletal muscle is a crucial regulator of glucose metabolism and lipid homeostasis [4,5], and it accounts for approximately 80% of insulin-mediated glucose uptake [6]. Therefore, skeletal muscle dysfunction is associated with metabolic diseases, such as type-2 diabetes [7]. Myogenesis mainly occurs in embryo, and it starts at E10.5 in PAX3-positive muscle progenitors for mouse, then myoblast cells are formed at E14.5 which could differentiate and fuse between themselves to give rise to myofibers driven by a list of transcription factors [8]. After birth, myofibers become enlarged and thicker to promote skeletal muscle growth [9]. In adult, satellite cells expressing PAX7 are quiet under normal circumstances, and activated after injury to repair the damaged muscle [10]. Hence, the mechanism study of skeletal muscle formation is necessary for agricultural development and disease therapy. Although the developmental process and regulatory mechanism has been well studied in skeletal muscle, most research focused on non-coding RNAs [11,12,13], and the causes and mechanisms of some biological phenomena during skeletal muscle formation are still unclear.

The pentose phosphate pathway (PPP) is a branch of glycolysis, which produces NADPH for various biological synthesis processes and scavenging of reactive oxygen species [14]. Glucose 6-P dehydrogenase (G6PD) is the first rate-limiting enzyme in PPP. In red blood cells, the PPP is the only source of NADPH, and G6PD deficiency as well as attenuated PPP activity results in red blood cells diseases, such as hemolytic anemia [15]. It is a generally acknowledged fact that PPP is not active and G6PD is undetectable in skeletal muscle; however, there is little known about the mechanism and function of G6PD down-regulation in skeletal muscle development to date.

In the current study, we explored the molecular mechanism of G6PD down-regulation and researched its role in skeletal muscle development. Interestingly, we found that histone acetylation and DNA methylation act together on the expression of G6PD. G6PD down-regulation plays no role in myogenic differentiation, but significantly promotes the sensitivity and response of skeletal muscle cells to insulin. These results complement the unclear gap of regulatory mechanisms during myogenesis, and also provide new sight to treat type-2 diabetes.

## 2. Results

### 2.1. G6PD Was Down-Regulated during Skeletal Muscle Formation

**Figure 1:**
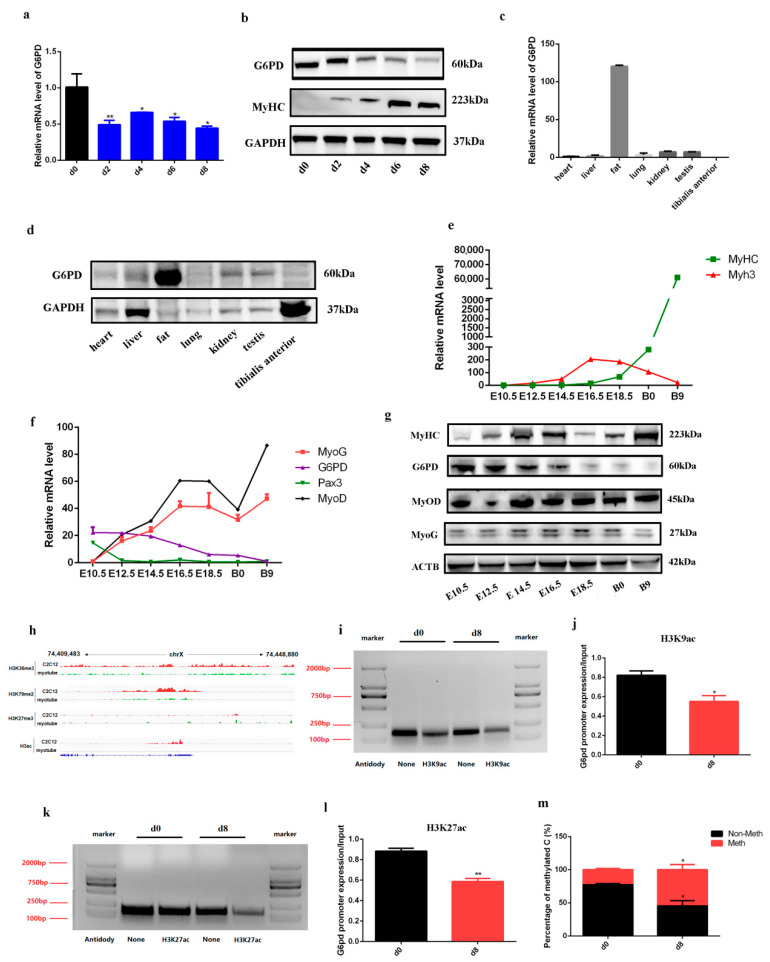
**G6PD was down−regulated during skeletal muscle formation.** (**a**) The transcriptional level of G6PD during C2C12 differentiation; data represent mean ± SD; *n* = 3; * *p* < 0.05, ** *p* < 0.01. (**b**) Western blot analysis for G6PD and MyHC during C2C12 differentiation. (**c**) The transcriptional level of G6PD in adult mice tissues; *n* = 3. (**d**) The protein level of G6PD in adult mice tissues. (**e**) The transcriptional level of MyHC and Myh3 during embryonic skeletal muscle formation; *n* = 3. (**f**) The transcriptional level of MyoD, MyoG, Pax3 and G6PD during embryonic skeletal muscle formation; *n* = 3. (**g**) Western blot analysis for MyHC, MyoD, MyoG and G6PD during embryonic skeletal muscle formation. (**h**) IGV graph shows the binding peak between G6PD promoter and H3K36me3, H3K79me2, H3K27me3 or H3ac. (**i**,**j**) Binding of H3K9ac to the G6PD promoter was detected by ChIP assays. DNA isolated from the precipitated complexes is the template for PCR, then PCR products were analyzed on a 1.5% agarose gel (**i**) and quantified by ImageJ (V1.8.0, National Institutes of Health, Berlin, Germany) (**j**); * *p* < 0.05. (**k**,**l**) The binding of H3K27ac to the G6PD promoter was detected by ChIP assays. PCR products were analyzed on a 1.5% agarose gel (**k**), and quantified by ImageJ (**l**); ** *p* < 0.01. (**m**) The percentage of DNA methylation and non-methylation for G6PD promoter was tested by BSP-seq and analyzed by SnapGene; *n* = 60; * *p* < 0.05. H3K36me3, histone H3 trimethylation at lysine 36; H3K79me2, histone H3 dimethylation at lysine 79; H3K27me3, histone H3 trimethylation at lysine 27; H3ac, histone H3 acetylation; d0, C2C12 myoblasts cultured in growth medium; myocyte, C2C12 cells cultured in differentiation medium for 80 h; d8, C2C12 cells cultured in differentiation medium for 8 d.

To explore the expression pattern of G6PD during myogenic differentiation, C2C12 cells were induced differentiation for 8 days after replacing growth medium (GM) with differentiation medium (DM). The result showed that mRNA level of G6PD was down-regulated (Figure 1a). MyHC is the marker of mature myotubes, and we found mature myotubes come into being at 2 days after differentiation, and its expression increased with the differentiation of C2C12 cells (Figure 1b); the protein level of G6PD was down-regulated, which is similar to its mRNA level (Figure 1b). Furthermore, we examined G6PD expression in adult mice tissues, and the results showed that both mRNA and protein of G6PD was undetectable in *tibialis anterior* muscle (Figure 1c,d). As muscle fibers begin to form in the embryonic period, and its number is basically stable after birth in mammalian [9,16,17], we collected mice embryos and tested G6PD expression during embryonic skeletal muscle formation. *Tibialis anterior* (TA) muscle can be harvested at E14.5. Myh3 is the marker of embryonic skeletal muscle, and we found it is significantly increased before E16.5. In addition, the expression of MyHC was increased (Figure 1e), and the expression of MyoG and MyoD, two important marker genes in skeletal muscle differentiation, was up-regulated; the expression of Pax3, a marker gene of skeletal muscle progenitor cells, was down-regulated during embryonic skeletal muscle formation (Figure 1f). In this process, G6PD decreased with the embryo develops (Figure 1f,g). At 9 days after birth, the mRNA and protein expression of G6PD were undetectable (Figure 1f,g). All these results indicated that G6PD was down-regulated during skeletal muscle formation.

To understand the mechanism of G6PD down-regulation, we downloaded CHIP-seq data of histone H3 trimethylation at lysine 36 (H3K36me3), histone H3 dimethylation at lysine 79 (H3K79me2), histone H3 trimethylation at lysine 27 (H3K27me3) and histone H3 acetylation (H3ac) from the ENCODE database, and explored the histone H3 modification of G6PD according to binding peak. As shown in Figure 1h, we found a strong binding between G6PD promoter and H3ac in C2C12 cells, and this binding was obviously down-regulated in myocyte cells (differentiation for 60 h); after blasting the peak region with mouse genome (*GRCm38.p6*) in NCBI, we designed two primers (mpG6PD-1 and mpG6PD-2) to amplify the peak region. The result showed that the product size of mpG6PD-1 is incorrect, and the product of mpG6PD-2 is correct (Figure S1a); next, we performed H3K9ac and H3K27ac CHIP experiment, and used mpG6PD-2 to test binding region in G6PD promoter. A total of 0.06 U/μL micrococcal nuclease had the best cutting efficiency (Figure S1b); the negative control indicated that there was no non-specific amplification for the CHIP experiment (Figure S1c). As shown in Figure 1i–l, after 8 days of differentiation, the binding of H3ac to G6PD promoter significantly decreased compared to C2C12 myoblast cells, indicating H3K9ac and H3K27ac were separated with G6PD promoter, which is in line with Figure 1h. In addition, bisulfite sequencing-PCR (BSP-seq) was performed to analyze whether the down-regulation of G6PD was regulated by DNA methylation. First, five BSP primers (BP) were designed to amplify G6PD promoter containing potential DNA methylation region in bisulfite-transformed mouse genome, and five control primers (CP) were used to amplify the same region in normal genome; the result showed that BP1 had no strip in normal mouse genome, while it had one strip in bisulfite-transformed mouse genome. At the same time, CP1 had one strip in normal mouse genome, while it had no strip in bisulfite-transformed mouse genome (Figure S1d), indicating the genome transformation by bisulfite is complete and BP1 is the most appropriate primer for BSP-seq. Next, C2C12 myoblast cells (d0) and myotubes (d8) were used to analyze DNA methylation of G6PD promoter during myogenic differentiation by BP1 primer. Finally, samples were tested by Sanger sequencing after verification by agarose gel electrophoresis (Figure S1e), and the results showed that the methylation level of G6PD promoter significantly increased after differentiation (Figure 1m). All these results illustrated that histone H3 acetylation and DNA methylation act together on the G6PD down-regulation during myogenic differentiation.

### 2.2. The Role of G6PD Deficiency in Skeletal Muscle Development

To explore the function of G6PD down-regulation during myogenic differentiation, we suspected that G6PD might be related to myogenesis. G6PD siRNAs were transfected into C2C12 myoblasts; at 24 h after transfection, C2C12 myoblast cells were induced differentiation for 3 days. As shown in Figure S2a, G6PD siRNAs significantly decreased the mRNA level of G6PD; however, MyoG, MyoD and MyHC were not changed by G6PD knock-down (Figure S2b–d). The protein expression is consistent with mRNA, and G6PD siRNAs significantly decreased G6PD protein level but not change the protein level of MyoG, MyoD and MyHC (Figure S2e). To further confirm this result, G6PD was over-expressed and the result showed that neither mRNA nor protein level of MyoG, MyoD and MyHC were changed after G6PD over-expression (Figure S2f–j). These results illustrated that G6PD deficiency is not necessary for skeletal muscle cells’ differentiation.

**Figure 2:**
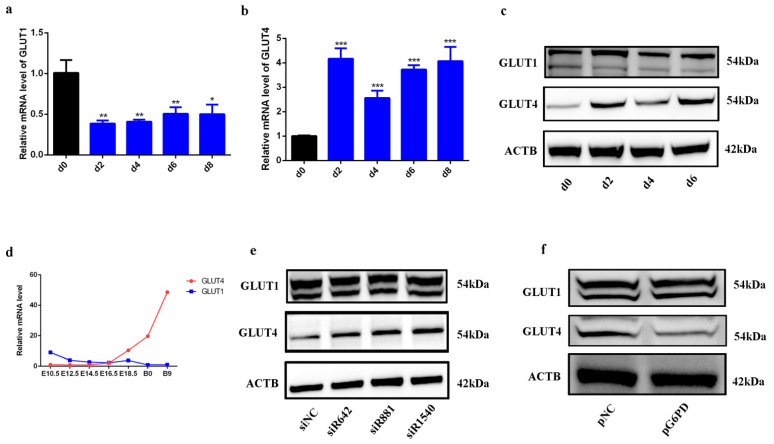
**Knock−down of G6PD promoted GLUT4 expression.** (**a**) The transcriptional level of GLUT1 during C2C12 differentiation; Data represent mean ± SD; *n* = 3. * *p* < 0.05, ** *p* < 0.01. (**b**) The transcriptional level of GLUT4 during C2C12 differentiation; data represent mean ± SD; *n* = 3. *** *p* < 0.001. (**c**) Western blot analysis for GLUT4 and GLUT1 during C2C12 differentiation. (**d**) The transcriptional level of GLUT1 and GLUT4 during embryonic skeletal muscle formation; *n* = 3. (**e**) Western blot analysis for GLUT4 and GLUT1 after G6PD siRNAs transfection. (**f**) The transcriptional level of GLUT4 and GLUT1 after G6PD over-expression; data represent mean ± SD; *n* = 3.

**Figure 3:**
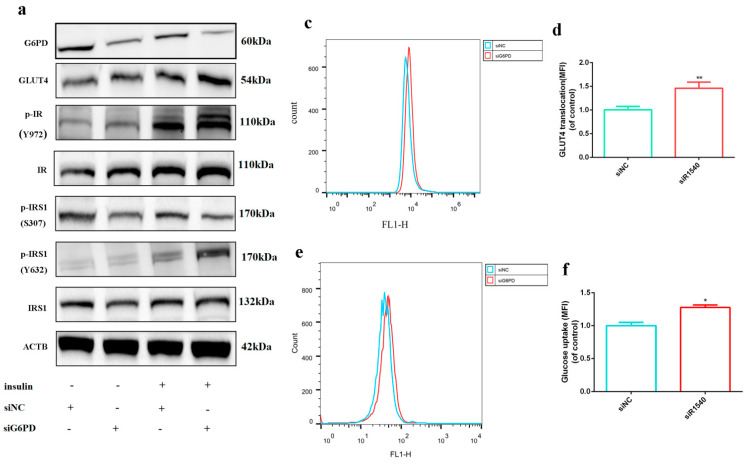

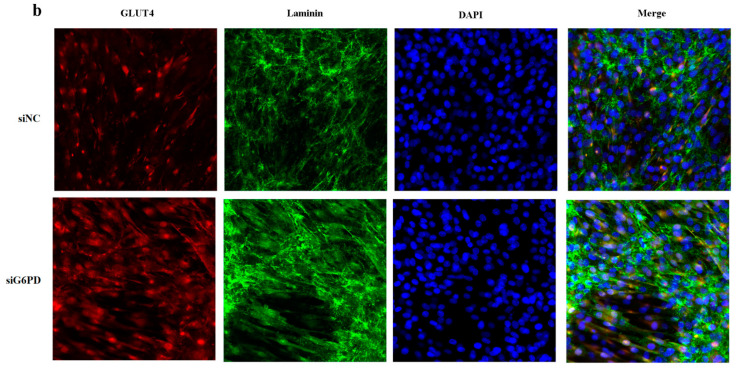
**Knock−down of G6PD promoted skeletal muscle cells’ response to insulin.** (**a**) Western blot analysis for G6PD, GLUT4 and insulin signaling pathway associated protein after G6PD siRNA transfection with or without 100 nM insulin. (**b**) Immunofluorescence assay of GLUT4 translocation on cell membrane after G6PD siRNA transfection under 100 nM insulin; GLUT4 was stained as red, Laminin was stained as green and cell nuclei were stained as blue; scale bar = 50 μm. (**c**,**d**) Flow cytometry assay of GLUT4 translocation on cell membrane after G6PD siRNA transfection under 100 nM insulin. (**e**,**f**) Flow cytometry assay for glucose uptake detection after G6PD siRNA transfection by 2-NBDG under 100 nM insulin. * *p* < 0.05, ** *p* < 0.01. MFI, median fluorescence intensity.

As G6PD is the first rate-limiting enzyme in pentose phosphate pathway (PPP), which is a branch of glucose metabolism [18], we next investigated whether G6PD is involved in the process of glucose metabolism. GLUT1 and GLUT4 are the most important glucose transporters in skeletal muscle, and we found GLUT1 decreased and GLUT4 increased after C2C12 differentiation (Figure 2a–c) or in embryonic skeletal muscle formation process (Figure 2d). GLUT4 was up-regulated by G6PD siRNAs transfection; consistently, GLUT4 was down-regulated after G6PD over-expression. GLUT1 was not changed after G6PD knock-down or over-expression (Figure 2e,f). GLUT4 is an insulin sensitive glucose transporter, and it transports from being intracellular to the cell surface under insulin stimulation [19,20,21]. The positive effect of G6PD deficiency on GLUT4 expression indicates its potential role in insulin signaling. As shown in Figure 3a, phosphorylation of insulin receptor (p^Y972^-IR) significantly increased after 100 nM insulin treatment, indicating insulin signaling was successfully activated by 100 nM insulin; after G6PD siR1540 transfection, G6PD was significantly down-regulated and GLUT4 was up-regulated; the phosphorylation of insulin receptor substrate 1 at Ser307 (p^S307^-IRS1), a negative mediator of insulin signaling, was significantly declined, while the phosphorylation of insulin receptor substrate 1 at Tyr632 (p^Y632^-IRS1), a positive mediator of insulin signaling, was significantly up-regulated. Since knock-down of G6PD can promote the activation of insulin signaling pathway, we next studied whether knock-down of G6PD has a positive effect on GLUT4 transport and glucose uptake under the stimulation of insulin. Immunofluorescence and flow cytometry assay of GLUT4 showed that knock-down of G6PD by G6PD siR1540 increased the expression of GLUT4 on cell membrane under 100 nM insulin (Figure 3b–d). A 2-NBDG assay suggested that knock-down of G6PD promoted glucose uptake (Figure 3e,f). All these results illustrated that knock-down of G6PD promoted skeletal muscle cells’ response to insulin.

### 2.3. Over-Expression of G6PD Decreased Insulin Signaling Activation

**Figure 4:**
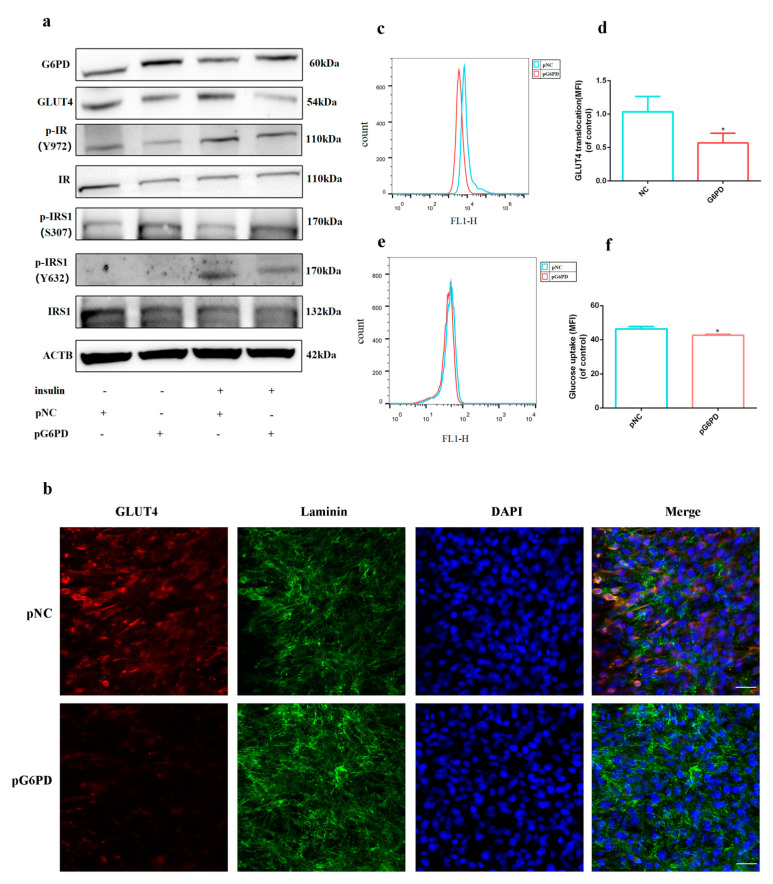
**Over−expression of G6PD impaired skeletal muscle cells’ response to insulin.** (**a**) Western blot analysis for G6PD, GLUT4 and insulin signaling pathway associated protein after G6PD over-expression with or without 100 nM insulin. (**b**) Immunofluorescence assay of GLUT4 translocation on cell membrane after G6PD over-expression under 100 nM insulin; GLUT4 was stained as red, Laminin was stained as green and cell nuclei were stained as blue; scale bar = 50 μm. (**c**,**d**) Flow cytometry assay of GLUT4 translocation on cell membrane after G6PD over-expression under 100 nM insulin. (**e**,**f**) Flow cytometry assay for glucose uptake detection after G6PD over-expression by 2-NBDG under 100 nM insulin. * *p* < 0.05.

Consistent with G6PD knock-down, p^Y972^-IR significantly increased after 100 nM insulin treatment, indicating insulin signaling was successfully activated; over-expression of G6PD increased G6PD and down-regulated GLUT4 expression; the expression of p^S307^-IRS1 was up-regulated, and p^Y632^-IRS1 was significantly down-regulated after G6PD over-expression (Figure 4a), indicating over-expression of G6PD blocked activation of insulin signaling. Immunofluorescence and flow cytometry assay of GLUT4 showed that over-expression of G6PD decreased translocation of GLUT4 on cell membrane under 100 nM insulin (Figure 4b–d). 2-NBDG assay suggested that over-expression of G6PD decreased glucose uptake (Figure 4e,f). All these results illustrated that over-expression of G6PD impaired skeletal muscle cells’ response to insulin. 

### 2.4. G6PD Is Involved in Impaired Insulin Signaling Activation Caused by HFD Treatment 

**Figure 5:**
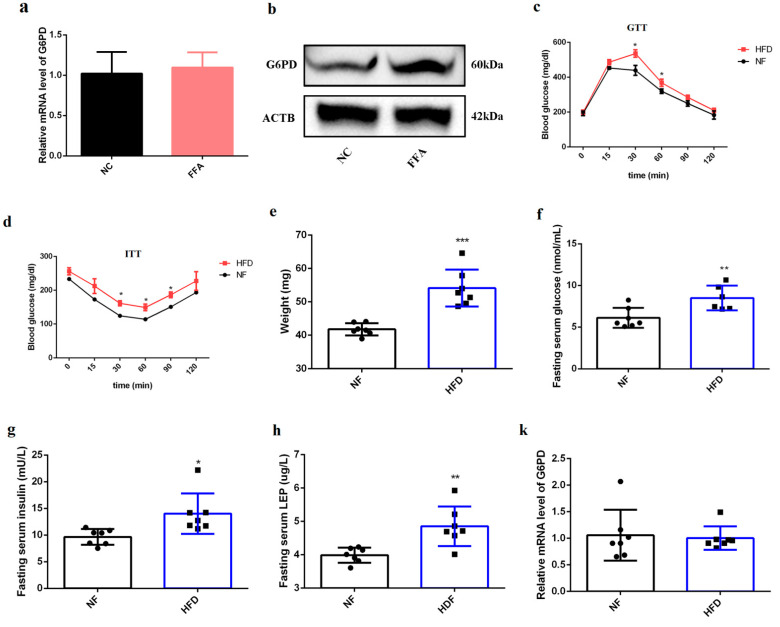

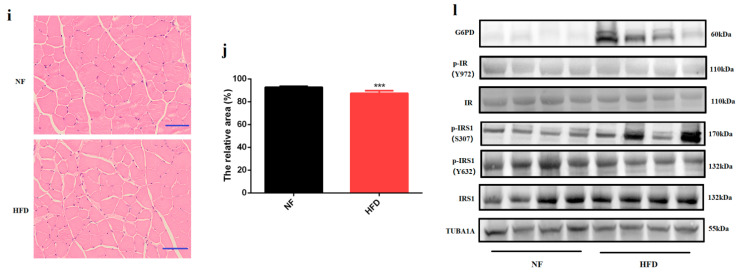
**G6PD is involved in impaired insulin signaling activation caused by HFD treatment.** (**a**) The transcriptional level of G6PD after FFA treatment; data represent mean ± SD; *n* = 3. (**b**) The protein level of G6PD after FFA treatment. (**c**,**d**) Blood glucose tolerant level in NF and HFD mice in response to glucose tolerant test (**c**) and insulin tolerant test (**d**). * *p* < 0.05. (**e**) The weight of mice fed by HFD for 8 weeks. *n* = 7. *** *p* < 0.001. (**f**) The fasting serum glucose in mice fed by HFD for 8 weeks. *n* = 7. ** *p* < 0.01. (**g**) The fasting serum insulin in mice fed by HFD for 8 weeks. *n* = 7. * *p* < 0.05. (**h**) The fasting serum leptin in mice fed by HFD for 8 weeks. *n* = 7. ** *p* < 0.01. (**i**,**j**) H&E staining of TA muscle in HFD mice (**i**), and the relative area was assessed by ImageJ (**j**). *n* = 3, scale bar = 100 µm. *** *p* < 0.001. (**k**) The transcriptional level of G6PD in HFD mice; data represent mean ± SD. (**l**) Western blot analysis for G6PD and insulin signaling associated proteins in HFD mice; *n* = 4. FFA, free fatty acid; NF, normal fed; HFD, high-fat diet; ITT, insulin tolerance test; GTT, glucose tolerance test; LEP, fasting serum leptin.

High-fat treatment resulted in insulin resistance characterized by an impaired insulin signaling pathway [22,23,24]. Since up-regulation of G6PD impaired insulin signaling response (Figure 4), we next explored whether high-fat treatment could result in insulin resistance by increasing G6PD. We found 1 mM of free-fatty acid (FFA) can establish an insulin-resistant cell model in previous study [25], so we tested the expression of G6PD after 1 mM FFA treatment; the result showed that the protein level of G6PD increased after FFA treatment, even though its mRNA level was not affected (Figure 5a,b). Meanwhile, after 8 weeks of high-fat diet (HFD) feeding, the mice weight was significantly increased (Figure 5c). Insulin tolerance test (ITT) and glucose tolerance test (GTT) results showed that glucose tolerance is impaired after HFD treatment (Figure 5d,e). In addition, fasting serum glucose, fasting serum insulin and fasting serum leptin (LEP) were significantly increased (Figure 5f–h); all these results indicate an insulin-resistant animal model was successfully built. The relative area of skeletal muscle in HFD mice was significantly down-regulated (Figure 5i,j). Protein expression of G6PD was significantly up-regulated in HFD-treated mice and its mRNA was not affected (Figure 5k,l), indicating G6PD is involved in impaired insulin signaling activation caused by HFD. 

### 2.5. Knock-Down of G6PD Rescued the Impaired Insulin Signaling in Insulin Resistant Model

**Figure 6:**
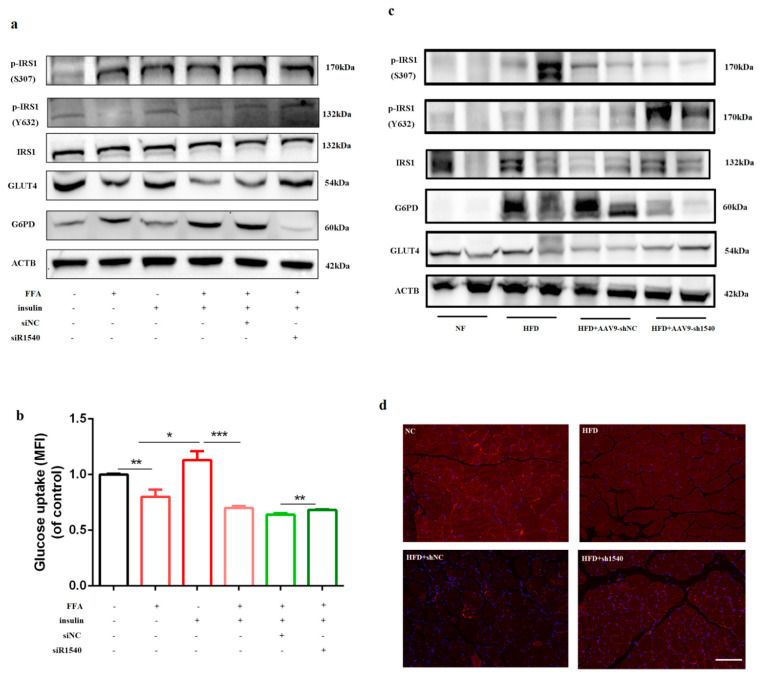
**Knock−down of G6PD rescued the impaired insulin signaling in an insulin-resistant model.** (**a**) Western blot analysis for G6PD, GLUT4 and insulin signaling protein after co-treatment with FFA, insulin and G6PD siRNA. (**b**) Flow cytometry assay for glucose uptake detection after co-treatment with FFA, insulin and G6PD siRNA. * *p* < 0.05, ** *p* < 0.01, *** *p* < 0.001. (**c**) Western blot analysis for G6PD, GLUT4 and insulin signaling protein after AAV9-sh1540 injection in HFD mice. (**d**) GLUT4 membrane translocation after AAV9-sh1540 injected HFD mice; scale bar = 100 μm.

As high-fat diet treatment impaired insulin signaling by increasing G6PD, we next explored whether knock-down of G6PD can rescued the impaired glucose uptake in insulin resistant cells. As shown in Figure 6a, FFA treatment increased G6PD and decreased GLUT4; meanwhile, p^Y632^-IRS1 was significantly down-regulated and p^S307^-IRS1 was significantly up-regulated. Compared to insulin group, co-treatment group of insulin and FFA increased G6PD and decreased GLUT4, and p^Y632^-IRS1 was significantly down-regulated. All these results illustrated FFA impaired insulin signaling. Transfection of G6PD siR1540 decreased G6PD and increased GLUT4; consistently, p^Y632^-IRS1 was up-regulated and p^S307^-IRS1 was significantly down-regulated. In addition, we found knock-down of G6PD significantly rescued the drop in glucose uptake caused by FFA compared to siNC (Figure 6b).

Then, insulin resistant mice fed by HFD for 8 weeks were injected AAV9-sh1540 for right legs and AAV9-shNC for left legs, which means for a HFD mouse, the left leg is the control group and the right leg is the treatment group; after 6 weeks, TA muscles were harvested to analysis. H&E staining indicates AAV9 injection resulted in obvious myogenic injury, and nuclei of myogenic fundal membrane moved inward (Figure S3). AAV9-sh1540 significantly decreased mRNA and protein expression of G6PD compared to AAV9-shNC, indicating knock-down of G6PD in vivo was successful (Figure 6c). We found the expression of GLUT4 and p^Y632^-IRS1 was up-regulated, and the expression of p^S307^-IRS1 was significantly down-regulated after AAV9-sh1540 delivery (Figure 6c). More importantly, AAV9-sh1540 significantly rescued the down-regulation of GLUT4 membrane translocation caused by HFD (Figure 6d). All these results indicated that knock-down of G6PD is beneficial to the response of insulin resistant cells to insulin whether in vivo or in vitro.

## 3. Discussion

Epigenetic modification is crucial to the regulation of gene expression, and DNA modification plays important role in several biological processes and diseases, such as embryogenesis, cell differentiation and aging [26,27,28,29]. DNA methylation and histone modification are the two main epigenetic mechanisms, and histone modification to H3 is the most well studied and characterized [30]. In this study, we found the expression of G6PD was down-regulated not only during C2C12 differentiation in vitro, but also during embryonic skeletal muscle formation in vivo; G6PD was completely silenced in skeletal muscle of adult mice. After analyzing the existing CHIP-seq data for H3 modification, we found that H3K36me3, H3K79me2 and H3ac, which usually induce transcriptional activation of genes, interact with the promoter of G6PD in C2C12 myoblasts; after 60 h differentiation, the binding between G6PD promoter and H3K36me3 or H3K79me2 modification is decreased, and the binding between G6PD promoter and H3ac has almost disappeared. Dhagia et al. found that G6PD activity contributes to the regulation of histone acetylation [31], and our CHIP result showed that histone acetylation is involved in regulating G6PD expression, indicating there are bidirectional regulation between G6PD and H3ac. BSP-Seq is regarded as the gold standard for DNA methylation detection [32], and our result showed that DNA methylation of G6PD promoter significantly increased after differentiation. It is well known that G6PD and PPP is absent in skeletal muscle; however, there is little known about its mechanism. This study explored how G6PD was down-regulated and complemented the unclear gap of regulatory mechanisms during myogenesis.

In previous studies, we found that G6PD is a target gene of miR-206, and miR-206 affects the C2C12 proliferation by down-regulating the protein expression of G6PD [33]. In addition, previous research has illustrated that G6PD deficiency is beneficial to MYH11 expression in smooth muscle cells [31]. Therefore, we first speculated that the down-regulation of G6PD could affect the differentiation of skeletal muscle. However, the results in Figure 2 showed that neither knock-down of G6PD nor over-expression of G6PD affects skeletal muscle differentiation. All these results illustrated that even though the expression of G6PD decreased during myogenesis, it is not a necessary factor for skeletal muscle formation. To date, the role of G6PD deficiency in skeletal muscle is unclear, and most research focused on skeletal muscle damage [34,35]. Our study provides explicit evidence for the function of G6PD on myogenic formation.

Since G6PD is a rate-limiting enzyme in the PPP, we wondered whether the down-regulation of G6PD is related to glucose metabolism in skeletal muscle cells. GLUT4 and GLUT1 are the two most important glucose transporters in skeletal muscle [36]. GLUT4 is sensitive to insulin, and researchers found oryzanol attenuates insulin resistance by increasing GLUT4 expression in skeletal muscle [37]. We found that GLUT1 is down-regulated and GLUT4 is up-regulated during differentiation. Skeletal muscle plays crucial role in glucose metabolism, both for its role in insulin response and its importance in exercise [38]; therefore, we think the up-regulation of GLUT4 is helpful for skeletal muscle’s response to insulin and exercise. Knock-down of G6PD promotes the expression of GLUT4 and the activation of insulin signaling, while over-expression of G6PD decreases GLUT4 and insulin signaling, illustrating G6PD deficiency is of great value for the insulin response in skeletal muscle.

In our previous study, miR-206 was found to be down-regulated after FFA treatment [25], and G6PD was also identified as a target gene of miR-206 [33]. Coinciding with these results, FFA increased G6PD protein expression in skeletal muscle cells, and this further suggests the positive role of G6PD deficiency in insulin signaling activation. In recent years, the incidence of type-2 diabetes has increased dramatically, and it has become one of the biggest public health problems in the world [39]. Insulin resistance is a major feature of type-2 diabetes, and it is manifested in the decreased response of insulin signaling pathway to insulin, which leads to the decreased glucose uptake in insulin-sensitive organs, such as skeletal muscle, fat and liver [39]. Exercise promotes glucose uptake by activating AMPK, which is an insulin-independent pathway in skeletal muscle [40]. Therefore, skeletal muscle is the main target to treat type-2 diabetes. Adeno-associated virus (AAV) is a non-enveloped virus which could deliver DNA to target tissues and cells, and AAV serotype 9 (AAV9) was the most efficient vector to deliver exogenous gene into skeletal muscle [41,42]. In this study, we constructed G6PD sh1540 into AAV9 vector, and injected AAV9-sh1540 into HFD mice; knock-down of G6PD in skeletal muscle rescued the impaired insulin signaling caused by HFD. To date, there is little known about the relationship between G6PD expression and insulin signaling activation’s stimulation in skeletal muscle, such as exercise and metformin; most research focused on its role in red blood cells. Ruggiero et al. found that the metformin, an oral antidiabetic agent, induced hemolytic anemia when G6PD was deficient [43], and Nikolaidis et al. found exercise did not affect the levels of oxidative stress in G6PD deficiency patients even though the level of GSH was significantly up-regulated [44]. However, how these factors influence G6PD in skeletal muscle still needs further studied.

## 4. Materials and Methods

### 4.1. Reagents and Antibodies

2-NBDG Glucose Uptake Assay Kit (Cell-Based, K682) was purchased from Biovision (Milpitas, CA, USA). Pierce agarose CHIP kit (26156) was purchased from ThermoFisher Scientific (Waltham, MA, USA). Cell-Light^TM^ EdU Apollo567 In Vitro Kit (C103110-1) was purchased from RiboBio (Guangzhou, China). Antibodies against H3K9ac (A7255) and H3K27ac (A2363) were purchased from ABclonal (Woburn, MA, USA). Antibodies against Myogenin (67082-1-Ig) and G6PD (66373-1-Ig) and GLUT1 (21829-1-AP) were purchased from proteintech (Wuhan, China). Antibody against myosin heavy chain (MyHC, MF20) was purchased from DSHB (Iowa city, IA, USA). Antibodies against GLUT4 (#2213) and MyOD1 (#13812) and mouse IgG1 isotype control (#5415) and Alexa Fluor^®^488 conjugate anti-mouse IgG (H + L) were purchased from Cell Signaling Technology (Beverly, MA, USA). Antibodies against GAPDH (ab9485) and p-IRS1 (S307, ab5599) and p-IRS1 (Y632, ab109543) and p-IR (Y972, ab5678) and IRS1 (ab52167) and IR (ab137747) and Laminin (ab11575) and Alexa Fluor^®^488 conjugate anti-rabbit IgG (H + L) (ab150077) were purchased from Abcam (Cambridge, UK). Ready-to-use 4′,6-diamidino-2-phenylindole (DAPI, KGA215-50) was purchased from KeyGEN BioTECH (Nanjing, China).

### 4.2. Animals

ICR mice that were 4 weeks old were purchased from the Qing Long Shan Animal Breeding Center (Nanjing, China) and housed with free access to water and food at room temperature. For high-fat treatment, high-fat diet (HFD) was used for 8 weeks and normal diet (NF) was used for the control group. Pregnant mice were slaughtered to obtain embryos at embryo 10.5 (E10.5), embryo 12.5 (E12.5), embryo 14.5 (E14.5), embryo 16.5 (E16.5) and embryo 18.5 (E18.5); neonatal mice were slaughtered on the day of birth (B0) and 9 days (B9) after birth. Mice were euthanized by cervical dislocation. The heart, liver, spleen, lungs, kidneys, muscle and fat were collected and stored in liquid nitrogen. All procedures were performed in accordance with the guidelines issued by the Animal Research Institute Committee of Nanjing Agricultural University (SYXK-2017-0027).

### 4.3. Cell Culture

The C2C12 mouse myoblast cell line was purchased from the Stem Cell Bank of the Chinese Academy of Sciences (Shanghai, China) and seeded into T25 flasks. These cells were maintained in growth medium (GM), which comprised Dulbecco’s modification of Eagle’s medium (DMEM, Hyclone Laboratories, Logan, UT, USA) containing 10% fetal bovine serum (FBS, Millipore, Sigma, Burlington, MA, USA), and differentiated in differentiation medium (DM), which comprised DMEM containing 2% horse serum (Millipore, Sigma). Free fatty acid (FFA) was dissolved in 5% bovine serum albumin (BSA; Millipore, Sigma) and its final concentration is 1 mM; insulin was purchased from Sigma (I9278, Sigma) and the final concentration is 100 nM. All cells were incubated at 37 °C in an atmosphere containing 5% CO_2_ and 95% air.

### 4.4. RNA Oligonucleotides and Cell Transfection

The G6PD siRNA and G6PD over-expression plasmid were purchased from GenePharma (Shanghai, China; Table S1). Transfection was performed using Lipofectamine 3000 reagent (ThermoFisher Scientific, Waltham, MA, USA) according to the manufacturer’s instructions.

### 4.5. RNA Extraction and Real-Time Quantitative PCR

Total RNA was extracted using TRIzol reagent (ThermoFisher Scientific). mRNAs were reverse transcribed into cDNA using PrimeScript RT master mix (TaKaRa Bio, Kusatsu, Japan). RT-qPCR was performed on a Step-One Plus real-time PCR system using AceQ qPCR SYBR green master mix (TaKaRa Bio). Relative expression levels were calculated using the 2^−ΔΔCT^ method. All primers were synthesized by Qingke (Beijing, China). The sequences of the primers are listed in Table S2.

### 4.6. Protein Extraction and Western Blot Analysis

Total protein was extracted using RIPA buffer (Applygen, Beijing, China) supplemented with 1 mM phenylmethylsulfonyl fluoride (PMSF; Beyotime, Shanghai, China). The protein concentration was determined using a BCA Protein Assay Kit (Beyotime). Western blotting was performed to investigate the protein expression. Protein denaturation was performed via boiling cell lysates for 10 min in SDS loading buffer (biosharp, Shanghai, China). Briefly, samples (each containing 30 μg protein) were electrophoresed on a 4–20% ExpressPlus PAGE Gel (GeneScript, Nanjing, China) and transferred to a PVDF membrane (Millipore, Sigma). The membrane was subsequently blocked with 5% bovine serum albumin (BSA, Millipore, Sigma) prepared in Tris-buffered saline containing Tween-20 (TBST) for 2 h at room temperature. Thereafter, the membrane was incubated overnight at 4 °C with diluted antibodies (1:1000 dilution) against Myogenin, MyHC, Myod1, p-IR (Y972), IR, p-IRS1 (S307), p-IRS1 (Y632), IRS1, GLUT1, GLUT4, ACTB and GAPDH. Next, the membrane was incubated with a HRP-conjugated goat anti-rabbit IgG antibody (1:2000, #7074; Cell Signaling Technology, Danvers, MA, USA) or anti-mouse IgG antibody (1:2000, ab190475; abcam) for 2 h at room temperature. Signals were visualized using WesternBright ECL Chemiluminescent HRP substrate (Advansta, Bering Drive, San Jose, CA, USA) and an ImageQuant LAS-4000 system (Fujifilm, Tokyo, Japan). Signal intensities were analyzed using ImageJ software.

### 4.7. Immunofluorescence Assay

Immunofluorescence assay was performed to determine membrane translocation of GLUT4. C2C12 cells were washed three times with PBS, and then fixed in 4% paraformaldehyde at ambient temperature for 1 h. Cells were permeabilized with 0.5% Triton X-100 at 4 °C for 10 min. Cells were immunostained with mixed antibodies containing rabbit antibody against Laminin and mouse antibody against GLUT4 overnight at 4 °C, followed by blocking in 1% BSA for 1 h. Next, cells were incubated for 2 h with mixed antibodies containing 647 goat anti-mouse IgG (H + L) and 488 goat anti-rabbit IgG (H + L). Finally, cell nuclei were stained with DAPI for 15 min. Fluorescent images were captured using a Zeiss LSM 710 META confocal microscope (LSM700, Carl Zeiss, Oberkochen, Germany).

### 4.8. Flow Cytometry

For 2-NBDG glucose uptake experiments, cells were plated in 12-well plates, and after G6PD siRNA or G6PD over-expression plasmid transfection, C2C12 cells were induced differentiation for 3 days with or without 1 mM FFA. Then, the treated cells were detached by trypsin and re-suspended in phosphate-buffered saline (PBS) containing 2-NBDG with or without insulin (100 nM) for another 30 min at 37 °C in the dark. Then, the cells were washed twice with cold washing buffer to remove free 2-NBDG and re-suspended in washing buffer. 

For GLUT4 translocation experiments, the cells were treated as a 2-NBDG experiment; then, the treated cells were detached by trypsin and re-suspended in PBS; next, antibodies against isotype control mouse (IgG1) or GLUT4 were added into cells for 2 h. Then, the cells were washed twice with PBS and re-suspended in 0.2 mL PBS. Next, cells were incubated with Alexa Fluor^®^ 488 Conjugate anti-mouse IgG (H + L) for 1 h. After being washed twice by PBS, cells were re-suspended in 0.2 mL PBS. Samples were analyzed with the BD Accuri C6 flow cytometer (Becton Dickinson, Franklin Lakes, NJ, USA) to 10,000 events per sample. The mean fluorescence intensity (MFI) served as a measure on a per cell basis.

### 4.9. Chromatin Immunoprecipitation (CHIP) Analysis

All CHIP samples were collected from 10 cm cell culture dishes. Briefly, cell pellet was isolated after treatment with 1% formaldehyde and 0.125 M glycine solution; then, micrococcal nuclease was used to digest chromatin at 0.06 U/μL concentration. For Immunoprecipitation, digested chromatin was mixed with H3K9ac or H3K27ac antibodies separately at 4 °C overnight. Finally, the purified DNA was collected after IP elution and DNA recovery, and PCR was conducted to detect the changes of target genes.

### 4.10. Bisulfite Sequencing PCR (BSP-Seq) Analysis

In total, 800 ng genome DNA was transformed by bisulfite. BSP primers were used to amplify the target region. When the stripe size is correct, PCR products were used to perform Sanger sequencing by Qingke (Beijing, China). The sequences of the BSP primers are listed in Table S2.

### 4.11. Adeno-Associated Virus Serotype 9 (AAV9)-Vector Construction and Administration

To generate G6PD-knockdown AAV9 vector, shRNA targeting G6PD or NC scramble sequences were compounded and inserted into AAV9 vector by GenePharma (Shanghai, China; Table S1). Virus titer was detected by qPCR; the titer of AAV9-shG6PD is 1.41 E + 13 V.G/mL, and the titer of AAV9-shNC is 7.21 E + 12 V.G/mL. For AAV9 gene delivery, 1.0 E + 13 V.G was injected into the *tibialis anterior* (TA) muscle of each mouse. AAV9-shNC was injected into the left leg, and AAV9-shG6PD was injected into the right leg; after 6-weeks administration, TA muscles were harvested for analysis.

### 4.12. Statistical Analysis

Statistical analyses were performed using Prism 6 software (GraphPad Software, La Jolla, CA, USA). Results are expressed as means ± SD, and error bars represent the SD of three replicates unless stated otherwise. Data were compared using the two-tailed unpaired Student’s *t* test and one-way ANOVA. The value of *p* < 0.05 was considered significant.

## 5. Conclusions

In summary, this study investigated the molecular mechanism of G6PD down-regulation and its function during skeletal muscle development process. We found that histone acetylation and DNA methylation act together on the regulation of G6PD. G6PD down-regulation plays no role in myogenic differentiation, but significantly promotes the sensitivity and response of skeletal muscle cells to insulin. All these results are of great significance for researching glucose metabolism and insulin response in skeletal muscle, which will provide new sight to treat type-2 diabetes. However, the molecular mechanism by which G6PD was involved in insulin signaling activation still needs further research.

## Supplementary Materials

The following supporting information can be downloaded at: https://www.mdpi.com/article/10.3390/ijms23137425/s1.
