# Supplementary material for: G6PD Deficiency Is Crucial for Insulin Signaling Activation in Skeletal Muscle
Source: Int J Mol Sci. 2022 Jul 4;23(13):7425. doi: 10.3390/ijms23137425 (PMC9267066; doi:10.3390/ijms23137425)
Supplement: Supplementary file 1 [file ijms-23-07425-s001.zip › ijms-1797468-supplementary.pdf]

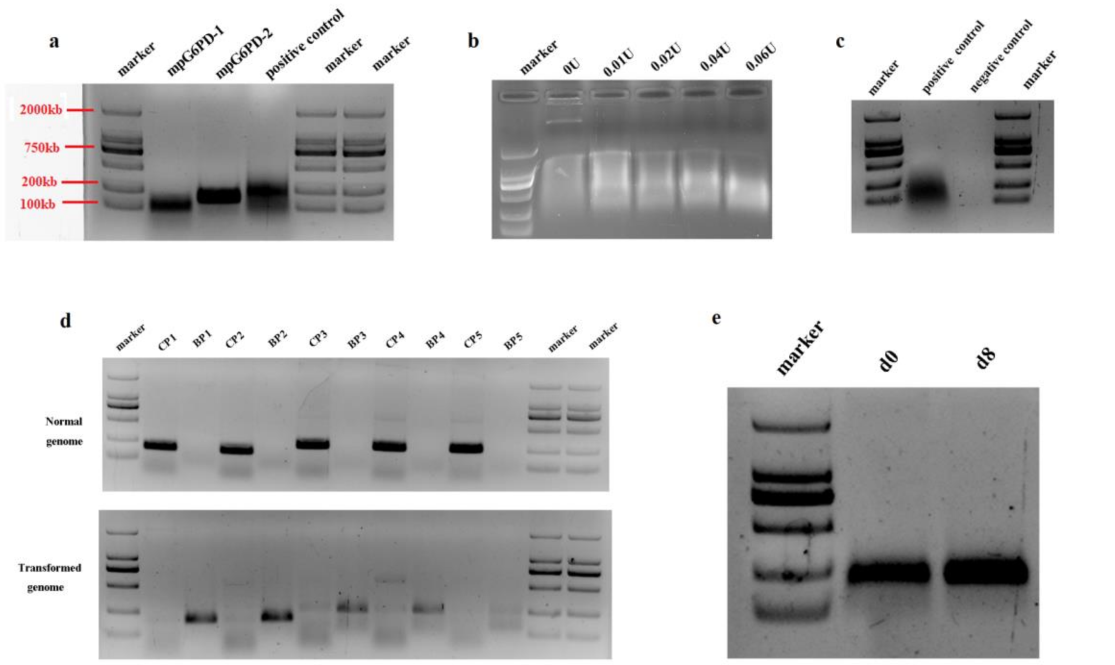

Figure S1. Exploration of CHIP and BSP-seq experimental conditions. a Primers validation which can amplify the binding region between H3ac and G6PD promoter. mpG6PD-1 and mpG6PD-2 were used to amplify the target region in mouse G6PD promoter; the expected size of mpG6PD-1 is 80bp ,and the expected size of mpG6PD-2 is 158bp. GAPDH primer was used as positive control to amplify GAPDH promoter in mouse genome. **b** Micrococcal nuclease was used to digest chromatin, and the working concentrations of micrococcal nuclease were shown in every column. **c** Anti-RNA Polymerase II Antibody and normal rabbit IgG were used as positive control and negative control respectively. **d** Primers selection for BSP-seq. BP were designed to amplify G6PD promoter containing potential DNA methylation region in bisulfite-transformed mouse genome, and CP were used to amplify the same region in normal genome. **e** Agarose gel verification before BSP-seq.

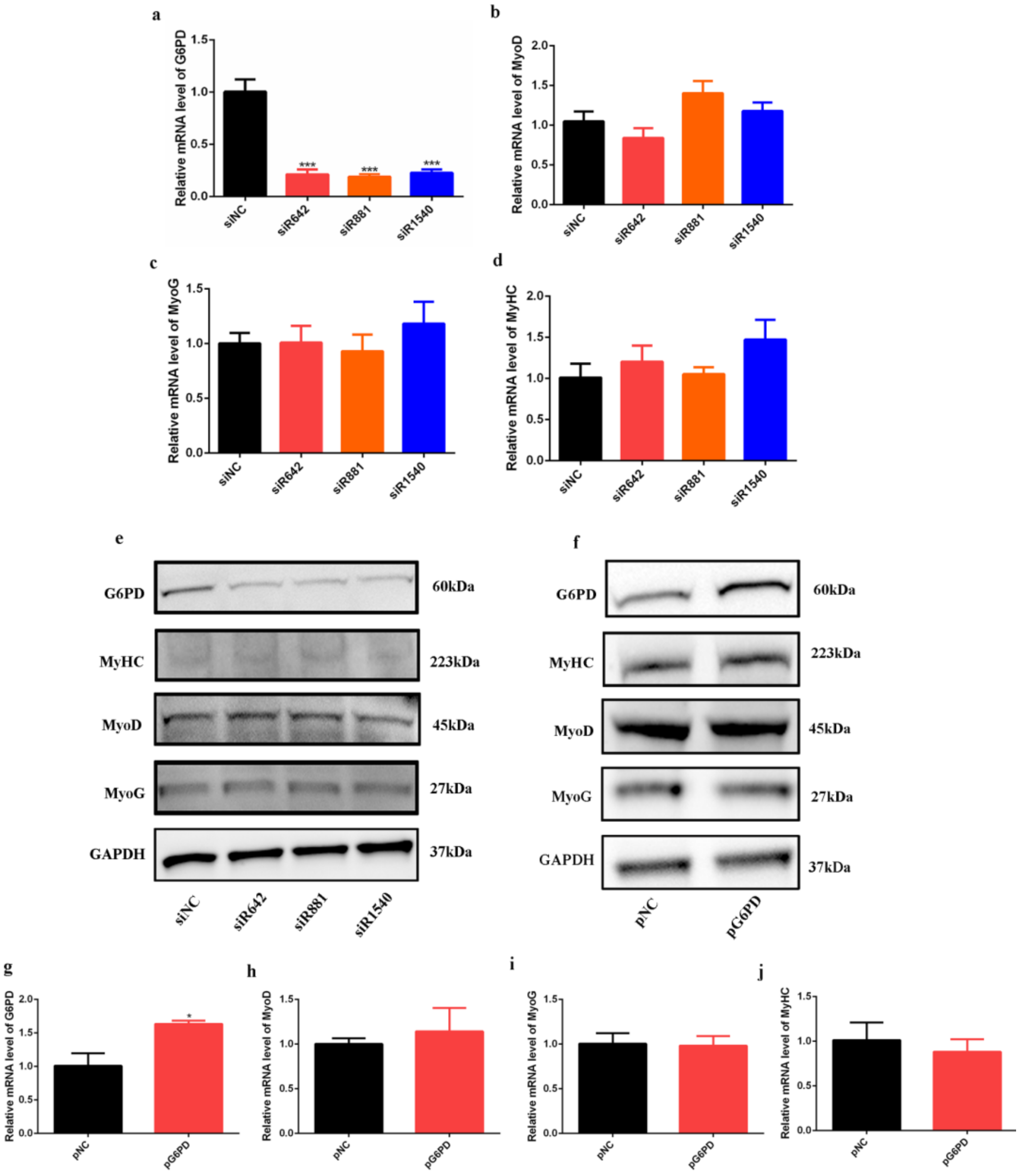

Figure S2. G6PD was not involved in myogenic differentiation. a The transcriptional level of G6PD after G6PD siRNAs transfection; Data represent mean ± SD; n = 3. ****P*< 0.001. **b** transcriptional level of MyoD after G6PD siRNAs transfection; Data represent mean ± SD; n = 3. **c** The transcriptional level of MyoG after G6PD siRNAs transfection; Data represent mean ± SD; n = 3. **d** The transcriptional level of MyHC after G6PD siRNAs transfection; Data represent mean ± SD; n = 3. **e** Western blot analysis for MyHC, MyoD, MyoG and G6PD after G6PD siRNAs transfection. **f** Western blot analysis for MyHC, MyoD, MyoG and G6PD after G6PD over-expression. **g** The transcriptional level of G6PD after G6PD over-expression; Data represent mean ± SD; n = 3. **P*< 0.05. **h** The transcriptional level of MyoD after G6PD over-expression; Data represent mean ± SD; n = 3. **i** The transcriptional level of MyoG after G6PD over-expression; Data represent mean ± SD; n = 3. **j** The transcriptional level of MyHC after G6PD over-expression; Data represent mean ± SD; n = 3.

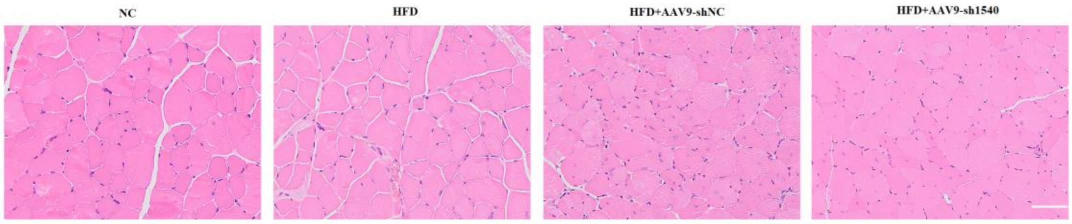

Figure S3. H&E staining of TA muscle in AAV9 sh1540 injected HFD mice. n=3 Scar bar = 100μm.

Table S1. Sequences of RNA Oligonucleotides.

Name		Sequence (5'-3')
Negative control	sense:	GGAGUUCUUUGCCCGUAAUTT
	anti-sense:	AUUCGGGCAAAGAACUCCTT
G6PD-mus-642	sense:	GGAGUUCUUUGCCCGUAAUTT
	anti-sense:	AUUACGGGCAAAGAACUCCTT
G6PD-mus-881	sense:	GCUCCAAUCAACUGUCGAATT
	anti-sense:	UUCGACAGUUGAUUGGAGCTT
G6PD-mus-1540	sense:	GCAGUAUACACCAAGAUGATT
	anti-sense:	UCAUCUUGGUGUAUACUGCTT
AAV9-shNC		ACTACCGTTGTTATAGGTG
AAV9-sh1540		GCAGTATACACCAAGATGA

Table S2. Primers sequences for qRT-PCR.

Gene name		Primer sequence (5'-3')
MyHC	F:	ACAGACATTTCCCAAATCCA
	R:	ATGTTCTTCTTCATCCGCTCC
Myh3	F:	TCCAAACCGTCTCTGCACTGTT
	R:	AGCGTACAAAGTGTGGGTGTGT
MyoG	F:	TGGAGCTGTATGAGACATCCC
	R:	TGGACAATGCTCAGGGGTCCC
MyOD	F:	GCCCGCGCTCCAACTGCTCTGAT
	R:	TCTTTTGGGCGTGAAGAACCAG

mpG6PD-1	F:	GTGGCTATGCTGCTAACGGA
	R:	TGTATGGCCTAGCTTTCCGC
mpG6PD-2	F:	GAAGCTACGAAACAGCCACC
	R:	AAGGGTGACCGCCTGACTA
BP-1	F:	ATAGTTGTAGTGAAGGGAAGGGTAGA
	R:	AATCCTCTTCTCCAAAAACAAAACT
BP-2	F:	TAGTTGTAGTGAAGGGAAGGGTAGAT
	R:	AATCCTCTTCTCCAAAAACAAAACT
BP-3	F:	TAGTTTGGTTTTGTTATTGGGTTTT
	R:	ACAACTCCACACAATTCTCCTAAAT
BP-4	F:	TTAGTTTGGTTTTGTTATTGGGTTT
	R:	ACAACTCCACACAATTCTCCTAAAT
BP-5	F:	TTTTAGTTTGGTTTTGTTATTGGGT
	R:	ACAACTCCACACAATTCTCCTAAAT
CP-1	F:	ACAGCTGCAGTGAAGGGAAGGGCAGA
	R:	GGTCCTCTTCTCCAAAGACAGGGCT
CP-2	F:	CAGCTGCAGTGAAGGGAAGGGCAGAT
	R:	GGTCCTCTTCTCCAAAGACAGGGCT
CP-3	F:	CAGTCTGGTCCTGCTACTGGGCTCT
	R:	GCAGCTCCACACAGTTCTCCTGGGT
CP-4	F:	TCAGTCTGGTCCTGCTACTGGGCTC
	R:	GCAGCTCCACACAGTTCTCCTGGGT
CP-5	F:	TTTCAGTCTGGTCCTGCTACTGGGC
	R:	GCAGCTCCACACAGTTCTCCTGGGT
GLUT4	F:	CCGCGGCCTCCTATGAGATACT
	R:	AGGCACCCCGAAGATGAGT
GLUT1	F:	GGCCTAAGGTCACATGAAGAAGG
	R:	AGCGGTGGTTCCATGTTTGA
ACTB	F:	ACAATGAGCTGCGTGTGGCC
	R:	CCTCGTAGATGGGCACAGTG
GAPDH	F:	ATCACTGCCACCCAGAAGACT
	R:	CATGCCAGTGAGCTTCCCGTT
G6PD	F:	GCCTCAGTGCTACTAGACATT
	R:	AGGGTTGGGATAGGAAAA

Note: mpG6PD primers amplify the promoter region of G6PD which was modified by H3ac in mouse genome; BP primers amplify the potential DNA methylation of G6PD promoter in bisulfite-transformed mouse genome; CP primers amplify the potential DNA methylation of G6PD promoter in normal mouse genome.
